# Plasma GFAP associates with secondary Alzheimer's pathology in Lewy body disease

**DOI:** 10.1002/acn3.51768

**Published:** 2023-03-31

**Authors:** Katheryn A. Q. Cousins, David J. Irwin, Alice Chen‐Plotkin, Leslie M. Shaw, Sanaz Arezoumandan, Edward B. Lee, David A. Wolk, Daniel Weintraub, Meredith Spindler, Andres Deik, Murray Grossman, Thomas F. Tropea

**Affiliations:** ^1^ Department of Neurology Perelman School of Medicine Philadelphia Pennsylvania USA; ^2^ Department of Pathology and Laboratory Medicine University of Pennsylvania Philadelphia Pennsylvania USA; ^3^ Department of Psychiatry University of Pennsylvania Philadelphia Pennsylvania USA

## Abstract

**Objective:**

Within Lewy body spectrum disorders (LBSD) with α‐synuclein pathology (αSyn), concomitant Alzheimer's disease (AD) pathology is common and is predictive of clinical outcomes, including cognitive impairment and decline. Plasma phosphorylated tau 181 (p‐tau_181_) is sensitive to AD neuropathologic change (ADNC) in clinical AD, and plasma glial fibrillary acidic protein (GFAP) is associated with the presence of β‐amyloid plaques. While these plasma biomarkers are well tested in clinical and pathological AD, their diagnostic and prognostic performance for concomitant AD in LBSD is unknown.

**Methods:**

In autopsy‐confirmed αSyn‐positive LBSD, we tested how plasma p‐tau_181_ and GFAP differed across αSyn with concomitant ADNC (αSyn+AD; *n* = 19) and αSyn without AD (αSyn; *n* = 30). Severity of burden was scored on a semiquantitative scale for several pathologies (e.g., β‐amyloid and tau), and scores were averaged across sampled brainstem, limbic, and neocortical regions.

**Results:**

Linear models showed that plasma GFAP was significantly higher in αSyn+AD compared to αSyn (*β* = 0.31, 95% CI = 0.065–0.56, and *P* = 0.015), after covarying for age at plasma, plasma‐to‐death interval, and sex; plasma p‐tau_181_ was not (*P* = 0.37). Next, linear models tested associations of AD pathological features with both plasma analytes, covarying for plasma‐to‐death, age at plasma, and sex. GFAP was significantly associated with brain *β*‐amyloid (*β* = 15, 95% CI = 6.1–25, and *P* = 0.0018) and tau burden (*β* = 12, 95% CI = 2.5–22, and *P* = 0.015); plasma p‐tau_181_ was not associated with either (both *P* > 0.34).

**Interpretation:**

Findings indicate that plasma GFAP may be sensitive to concomitant AD pathology in LBSD, especially accumulation of β‐amyloid plaques.

## Introduction

Lewy body spectrum disorders (LBSD) are a group of movement disorders that include Parkinson's disease (PD), PD with dementia (PDD), and dementia with Lewy bodies (DLB). While α‐synuclein (αSyn) is the primary pathology associated with LBSD, concomitant Alzheimer's disease (AD) pathology is common. Nearly 50% of cases have significant accumulations of β‐amyloid plaques and tau neurofibrillary tangles (intermediate or high AD neuropathologic change [ADNC]), justifying a secondary diagnosis of AD at autopsy.^1^, ^2^ Concomitant AD may describe much of the clinical heterogeneity across LBSD,^3^ and is associated with memory and linguistic impairments,^4^, ^5^, ^6^ postural instability and gait,^7^ and reduced survival.^2^, ^6^ Thus, the in vivo detection of concomitant AD is important for prognosis and disease management of LBSD^8^ and may inform clinical trial design.^9^ Yet, biofluid markers that are well established in canonical AD must still be validated in LBSD to test sensitivity to concomitant ADNC. Two factors may affect biomarker accuracy to detect secondary AD. First, overall burden of ADNC is relatively less severe in LBSD compared to AD, with high proportions of intermediate ADNC.^1^, ^2^ Second, cerebrospinal fluid (CSF) biomarkers of AD have an altered profile in LBSD. In particular, CSF tau phosphorylated at threonine 181 (p‐tau_181_) is inversely associated with αSyn pathology, which can reduce its sensitivity to concomitant AD.^10^ Another study shows that CSF p‐tau_181_ is significantly lower in early PD than controls, further evincing that concentrations are reduced in LBSD.^11^ Thus, as plasma biomarkers for AD are developed, it becomes important to validate their efficacy to detect AD copathology in LBSD.

Two candidate AD plasma biomarkers in LBSD are plasma p‐tau_181_ and glial fibrillary acidic protein (GFAP). Several studies in autopsy and living participants have shown that plasma p‐tau_181_ can detect AD^12^, ^13^ and is associated with accumulation and spread of brain β‐amyloid and tau pathology.^13^ GFAP is a cytoskeletal filament protein highly expressed in astrocytes, and concentrations of GFAP are raised in the CSF and blood following astrogliosis and the degeneration of astrocytes.^14^ Neuroinflammation plays a key role in AD pathogenesis,^15^ and plasma GFAP may be an early marker correlated with brain β‐amyloid pathology,^16^, ^17^ as well as white matter disease.^18^, ^19^ Still, the majority of work has studied both analytes in the context of primary AD. Utility of these plasma biomarkers in LBSD is unclear, due to different findings across studies of living LBSD, which define groups using clinical or positron emission tomography (PET) data.^20^, ^21^, ^22^ In light of these conflicting findings in biomarker‐defined LBSD, it becomes necessary to test plasma biomarkers in autopsy cases with pathologically confirmed diagnoses.

In this autopsy study, we compare plasma p‐tau_181_ and GFAP in αSyn with concomitant AD (αSyn+AD) versus αSyn without AD; AD without concomitant αSyn is included as a reference group. Multivariable models test how each analyte correlates with brain accumulation of pathological *β*‐amyloid plaques and tau neurofibrillary tangles, as well as αSyn deposition and gliosis. Receiver operating characteristic (ROC) analyses test how accurately analytes detect ADNC in this mixed pathology sample, and we also tested associations with global cognition (mini mental state exam [MMSE]).

## Methods

### General selection criteria

Participants were enrolled at the University of Pennsylvania (Penn) Parkinson's Disease Research Center, Frontotemporal Degeneration Center, or Alzheimer's Disease Research Center, and were selected retrospectively from the Penn Integrated Neurodegenerative Disease Database (INDD)^23^ on September 15, 2022, based on eligibility criteria outlined below. The Penn Institutional Review Board approved these studies and written informed consent was obtained from each participant.

Selection criteria were clinically diagnosed LBSD with neuropathologic diagnoses of either αSyn (*n* = 30) or αSyn+AD (*n* = 19), and available biomarkers of either plasma GFAP or plasma p‐tau_181_. As a neuropathologic reference group, we also examined autopsy‐confirmed AD without αSyn (*n* = 21), with a clinical diagnosis of AD, and available plasma GFAP and p‐tau_181_. If individuals had plasma collected at two or more timepoints, the last timepoint was selected to more closely reflect pathology at autopsy. There was a median interval of 2 years (interquartile range [IQR] = 2; max = 11) between plasma collection and death. Finally, we used propensity score matching^24^ to select 70 clinically normal individuals as controls, matched for age and sex, with an MMSE ≥ 27; controls did not have autopsy data.

### Neuropathologic diagnoses and assessments

Brains were sampled at autopsy and assessed for ADNC and αSyn, as well as for other pathologies according to standardized procedures.^23^, ^25^ ADNC was scored according to ABC criteria^26^ and high or intermediate ADNC was considered AD positive; low or not ADNC was considered AD negative. Thal phase,^27^ Braak stage,^28^ and CERAD score^29^ are reported using a 4‐point scale (0–3).^26^ DLB stage was assessed^30^ and brainstem predominant, limbic, and neocortical Lewy bodies were all considered αSyn positive; no or amygdala‐predominant Lewy bodies were considered αSyn negative. In addition to ADNC and αSyn, the presence of concomitant vascular disease^31^ and TDP‐43^32^ was assessed; only three LBSD patients total had moderate or high vascular disease (one αSyn+AD; two αSyn)^31^ and it was therefore not assessed in analyses.

Brain tissue samples were stained using immunohistochemistry as previously described,^23^ and gross severity of pathological accumulations of β‐amyloid, tau, αSyn, and TDP‐43 were scored using a semiquantitative scale (0 = none, 0.5 = rare, 1 = minimal, 2 = moderate, and 3 = severe); in addition, severity of gliosis and cerebral amyloid angiopathy (CAA) were likewise quantified. Burden scores were the average across regions standardly sampled,^26^ which included the amygdala, cingulate, CA1/subiculum, entorhinal cortex, middle frontal gyrus, angular gyrus, superior/middle temporal gyrus, pons, and medulla. Hemisphere was randomized; if both hemispheres were sampled, the average was taken. Missing regional data were dropped to calculate the average.

### Plasma analysis

Plasma was collected and assayed for p‐tau_181_ and GFAP previously described.^33^, ^34^ Plasma samples were analyzed on the Quanterix HD‐X automated immunoassay platform. Samples were analyzed in duplicate using the Discovery kit reagents for GFAP^35^ and using the V2 Advantage kit for p‐tau_181_.^33^

In our sample, one αSyn and one AD were missing plasma GFAP.

### Clinical and demographic features

Demographic features were available through INDD. Where applicable, we examined age at onset (first reported symptom; years), age at plasma collection (years), disease duration at plasma (time from onset to plasma collection; years), interval from plasma‐to‐CSF (years), interval from plasma‐to‐MMSE (years), interval from plasma‐to‐death (years), and age at death (years). Sex and race were determined by self‐report.

MMSE was used as a measure of global cognition in LBSD that was available in historical autopsy cases. In the LBSD autopsy sample, 24 αSyn and 16 αSyn+AD patients had MMSE available. There was a median interval of 0.6 years (IQR = 2; max = 8.2) between plasma collection and MMSE.

### Statistical analyses

Not all demographic and analyte variables were normally distributed, therefore, nonparametric Kruskal–Wallis and Mann–Whitney–Wilcoxon tests performed unadjusted group comparisons for continuous variables. Fisher's exact tests performed group comparisons for categorical variables; for larger contingency tables where Fisher's exact test was not able to be computed (e.g., DLB type and clinical diagnosis), Chi‐square tests were used. Spearman correlations tested within‐group associations between continuous variables; given the complex and interrelated relationship of pathological variables, both nominal and Bonferroni‐corrected *P*‐values are reported. All statistical models used a significance threshold of *α* = 0.05.

In addition to unadjusted comparisons and correlations, multiple regression was performed to control for possible confounds.^36^ For linear models, 95% confidence intervals (CI) for β‐estimates were reported. Plasma p‐tau_181_ and GFAP were not normally distributed and were log‐transformed in all models. Distributions of pathological and clinical measures varied and were therefore rank‐transformed to perform nonparametric analyses. Effect sizes with 95% CI were calculated using generalized *η* ^2^ (*η* ^2^_*G*_),^37^ using standard interpretation cutoffs (≥0.01 small, ≥0.06 medium, and ≥0.14 large).^38^ Statistical analyses were performed and figures were generated using R version 4.1.2 (2021‐11‐01).

First, linear models tested how plasma biomarkers differed across αSyn and αSyn+AD, covarying for age at plasma collection, interval from plasma‐to‐death, and sex (Equation 1).

$$\log\left(\text{PlasmaAnalyte}\right)=\beta_{0}+\beta_{1}\times \text{Group}+\beta_{2}\times \mathrm{Age}+\beta_{3}\times \text{Plasma‐to‐Death}+\beta_{4}\times \mathrm{Sex}+\varepsilon$$

Within LBSD, linear models tested how AD pathological hallmarks β‐amyloid plaques (Equation 2) and tau neurofibrillary tangles (Equation 3), associated with plasma GFAP and plasma p‐tau_181_, covarying for age, interval from plasma‐to‐death, and sex. Models were repeated excluding individuals with a plasma‐to‐death interval >5 years.

$$\text{rank}\left(\text{β‐amyloid}\right)=\beta_{0}+\beta_{1}\times \log\left(\text{p‐}\text{ta}{\text{u}}_{\text{181}}\right)+\beta_{2}\times \log\left(\text{GFAP}\right)+\beta_{3}\times \mathrm{Age}+\beta_{4}\times \text{Plasma‐to‐Death}+\beta_{5}\times \mathrm{Sex}+\varepsilon .$$

$$\mathrm{rank}\left(\mathrm{Tau}\right)=\beta_{0}+\beta_{1}\times \log\left(\text{p‐}\text{ta}{\text{u}}_{\text{181}}\right)+\beta_{2}\times \log\left(\text{GFAP}\right)+\beta_{3}\times \mathrm{Age}+\beta_{4}\times \text{Plasma‐to‐Death}+\beta_{5}\times \mathrm{Sex}+\varepsilon .$$

Given correlations between β‐amyloid, tau, and gliosis burden in LBSD, a post hoc model tested associations of all with plasma GFAP (Equation 4), covarying for age, interval from plasma‐to‐death, and sex.

$$\log\left(\text{GFAP}\right)=\beta_{0}+\beta_{1}\times \mathrm{rank}\left(\text{β−amyloid}\right)+\beta_{2}\times \mathrm{rank}\left(\mathrm{Tau}\right)+\beta_{3}\times \mathrm{rank}\left(\text{Gliosis}\right)+\beta_{4}\times \mathrm{Age}+\beta_{5}\times \text{Plasma‐to‐Death}+\beta_{6}\times \mathrm{Sex}+\varepsilon .$$

ROC analyses using bootstrapping (500 iterations) compared discrimination of high/intermediate ADNC from not/low ADNC; AUC with 90% CIs were reported.^39^ ROC analyses tested plasma analytes in the full autopsy sample (αSyn, αSyn+AD, and AD) and within LBSD (αSyn and αSyn+AD).

Finally, we tested associations of global cognition (MMSE, rank‐transformed) with both analytes, covarying for age, MMSE‐to‐plasma interval (years), and sex. This analysis was repeated excluding individuals with a plasma‐to‐MMSE interval >1 year.

$$\mathrm{rank}\left(\text{MMSE}\right)=\beta_{0}+\beta_{1}\times \log\left(\text{GFAP}\right)+\beta_{2}\times \log\left({\text{p‐tau}}_{\text{181}}\right)+\beta_{3}\times \mathrm{Age}+\beta_{4}\times \text{MMSE‐to‐plasma}+\beta_{5}\times \mathrm{Sex}+\varepsilon .$$

## Results

Table 1 summarizes group characteristics and Kruskal–Wallis groupwise comparisons. Pairwise Wilcoxon tests show that αSyn+AD had an older median age of onset than αSyn (W = 137.5, *P* = 0.0025); there was no difference in age at plasma (*P* = 0.37), plasma‐to‐death interval (*P* = 0.76), or age at death (*P* = 0.37). Fisher's exact tests showed no difference in sex distribution (*P* = 0.32); all LBSD individuals self‐identified as White. Compared to AD, αSyn+AD had lower Thal Phase (OR = 8.76, CI = 0.9–444.13, *P* = 0.04), Braak Stage (OR = 38.32, CI = 4.31–1904.9, *P* = 3.0e‐05), and CERAD score (*P* = 0.012). The proportion of APOE ε4 alleles did not differ between AD and αSyn+AD (*P* = 0.41).

**Table 1:** Demographic, pathological, and clinical characteristics of participants.

	Control	αSyn	αSyn+AD	AD	*p*
*n*	70	30	19	21	
Age at onset (years)	–	60 (54–66)	69 (62–74)	62 (59–66)	0.009
Age at plasma (years)	72 (65–77)	75 (68–79)	74 (70–84)	71 (64–75)	0.083
Duration at plasma (years)	–	13 (9–16)	7 (4–10)	5 (4–8)	<0.001
Plasma‐to‐death (years)	4 (2–5)	2 (1–2)	2 (1–2)	3 (2–4)	0.095
Age at death (years)	73 (70–79)	77 (70–81)	76 (72–84)	73 (69–77)	0.233
Sex = male (%)	39 (56%)	21 (70%)	16 (84%)	11 (52%)	0.074
Self‐reported race (%)					
Asian	2 (3%)	0 (0%)	0 (0%)	0 (0%)	0.004
Black or African American	16 (23%)	0 (0%)	0 (0%)	2 (10%)	
More than one race	1 (1%)	0 (0%)	0 (0%)	1 (5%)	
White	50 (72%)	30 (100%)	19 (100%)	18 (86%)	
Thal phase (0–3)	–	1 (0–1)	3 (2–3)	3 (3–3)	<0.001
Braak stage (0–3)	–	1 (1–2)	2 (2–3)	3 (3–3)	<0.001
CERAD score (0–3)	–	0 (0–1)	2 (2–3)	3 (3–3)	<0.001
ADNC (%)					
Not	–	11 (37%)	0 (0%)	0 (0%)	<0.001
Low	–	19 (63%)	0 (0%)	0 (0%)	
Intermediate	–	0 (0%)	14 (74%)	1 (5%)	
High	–	0 (0%)	5 (26%)	20 (95%)	
DLB type (%)					
None	–	0 (0%)	0 (0%)	10 (48%)	<0.001
Amygdala predominant	–	0 (0%)	0 (0%)	11 (52%)	
Brainstem predominant	–	6 (20%)	1 (5%)	0 (0%)	
Transitional or limbic	–	15 (50%)	5 (26%)	0 (0%)	
Diffuse or neocortical	–	9 (30%)	13 (68%)	0 (0%)	
Vascular disease+ (%)	–	2 (7%)	1 (5%)	3 (14%)	0.646
APOE ε4 (%)					
0	49 (72%)	19 (63%)	6 (33%)	6 (29%)	<0.001
1	17 (25%)	11 (37%)	10 (56%)	9 (43%)	
2	2 (3%)	0 (0%)	2 (11%)	6 (29%)	
Clinical diagnosis (%)					
Control	70 (100%)	0 (0%)	0 (0%)	0 (0%)	<0.001
AD	0 (0%)	0 (0%)	0 (0%)	18 (95%)	
PD/PDD	0 (0%)	27 (90%)	11 (58%)	0 (0%)	
DLB	0 (0%)	3 (10%)	8 (42%)	1 (5%)	

*Note*: For continuous variables, median and interquartile range (IQR) are reported; Kruskal–Wallis tests performed group comparisons. For categorical variables, count (percentage [%]) are provided; Fisher's exact tests performed frequency comparisons, except for larger contingency tables (DLB Type and Clinical Diagnosis) where Chi‐square tests were used. *P*‐values are reported for group comparisons. Note that DLB type and ADNC (including Thal, Braak, and CERAD) were used to define groups.

### Group comparisons

**Figure 1:**
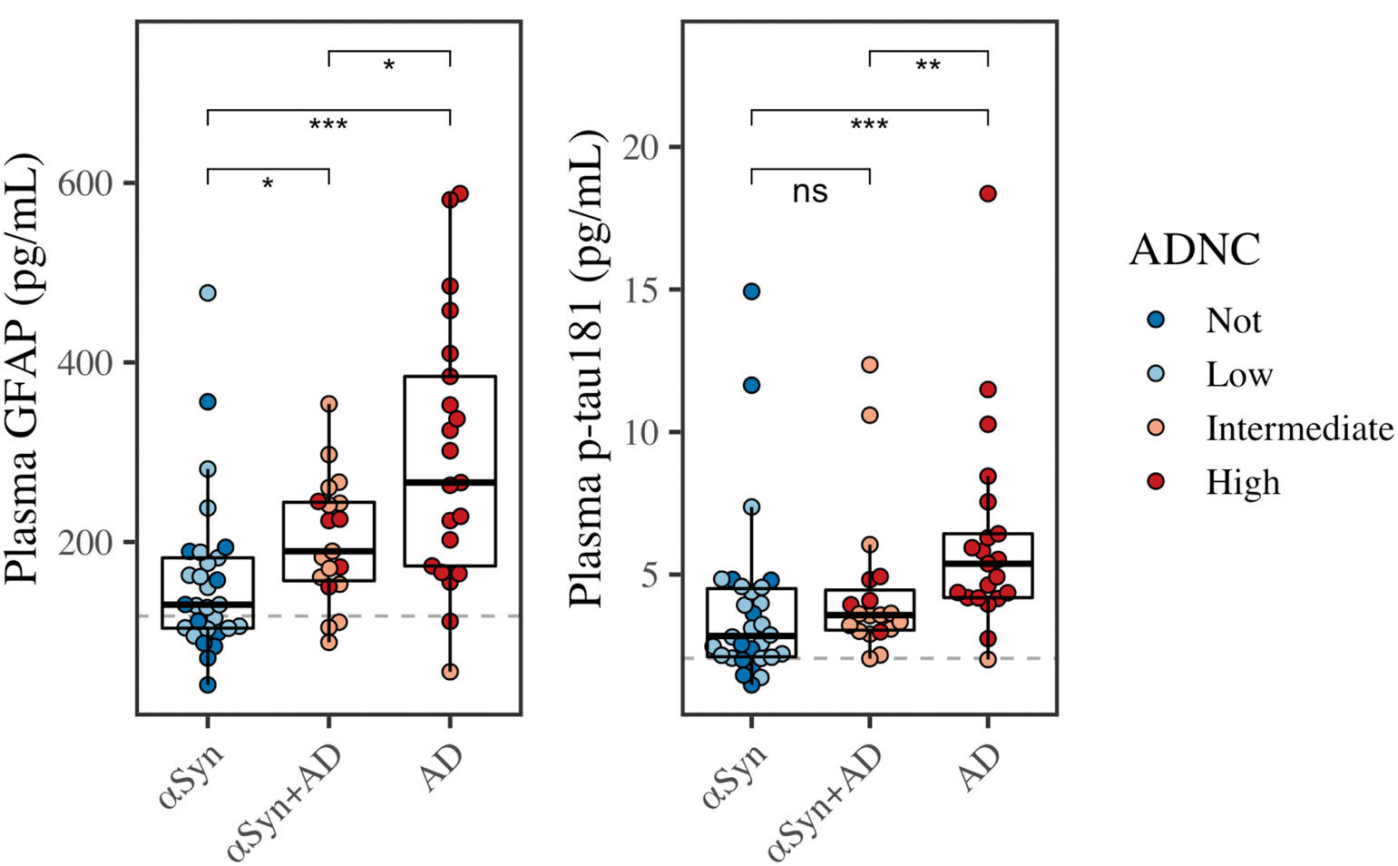
Plasma concentrations across pathological diagnosis. Boxplots show median, interquartile range (IQR), and outliers for plasma p‐tau_181_, GFAP, and NfL. Color represents ADNC. Horizontal dashed lines plot medians for GFAP and p‐tau_181_ from neurologically normal controls. Asterisks represent *P*‐values from Wilcoxon pairwise comparisons (*P < 0.05, **P < 0.01, ***P < 0.001, and not significant [ns]).

To test which plasma analytes associate with concomitant AD pathology, Figure 1 compares analyte levels across αSyn and αSyn+AD, with AD as a reference group. Linear models covarying for age at plasma, plasma‐to‐death interval, and sex confirmed that GFAP was significantly higher in αSyn+AD than αSyn (*β* = 0.31, 95% CI = 0.065–0.56, *P* = 0.015) with large effect size (*η* ^2^_*G*_ = 0.14; 95% CI = 0.021–1.0); plasma p‐tau_181_ was not significantly higher in αSyn+AD than αSyn (*P* = 0.37).

Examining covariates, age at plasma was positively associated with GFAP (*β* = 0.017, 95% CI = 0.00094–0.034, *P* = 0.039) but not p‐tau_181_ (*P* = 0.27). Plasma‐to‐death was inversely associated with GFAP (*β* = −0.056, 95% CI = −0.10 to −0.0078, *P* = 0.024) but not p‐tau_181_ (*P* = 0.61). Sex was associated with neither GFAP (*P* = 0.16) nor p‐tau_181_ (*P* = 0.12).

Compared to neurologically healthy controls, Wilcoxon tests showed that plasma GFAP was higher in αSyn+AD (W = 229, *P* = 0.000013) and AD groups (W = 174, *P* = 1.3e‐07), but not αSyn (W = 761.5, *P* = 0.052). Compared to controls, plasma p‐tau_181_ was higher in all three groups: αSyn+AD (W = 230.5, *P* = 0.000014), AD (W = 125, *P* = 9.4e‐09), and αSyn (W = 623, *P* = 0.0013).

Five patients with high/intermediate ADNC (αSyn+AD, AD) had plasma GFAP below the control median (117.6 pg/mL; see Figure 1). We tested if AD pathology individuals with low GFAP (<117.6) differed from AD with high GFAP values (>117.6). Low GFAP was associated with a significantly longer plasma‐to‐death interval (median = 9 [2]) than high GFAP (median = 2 [2]) (W = 30, *P* = 0.017). Low GFAP was also associated with a clinical diagnosis of PD/PDD (three [75%] PD/PDD; one [25%] DLB; zero AD) more than high GFAP (eight [24%] PD/PDD, eight [24%] DLB, and 18 [53%] AD) (Fisher's test: *P* = 0.039). There were no significant differences between high and low GFAP for age (*P* = 0.15), sex (*P* = 0.15), or APOE ε4 (*P* = 0.39), but we note the small sample size for low GFAP (*n* = 5).

**Figure 2:**
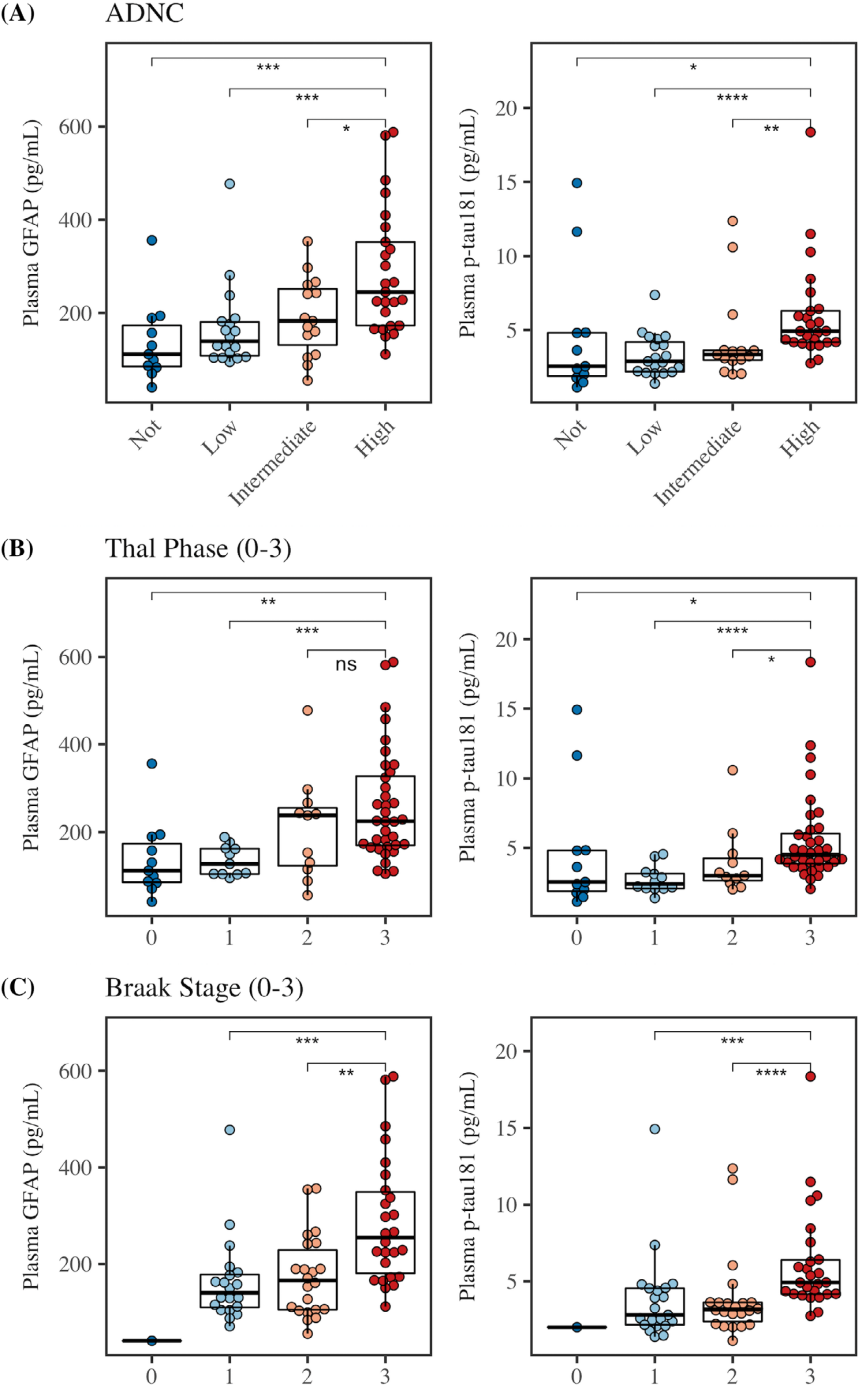
Plasma concentrations across ADNC, Thal Phase, and Braak Stage across all autopsy cases (αSyn, αSyn+AD, AD). Plasma GFAP (left panels) and p‐tau_181_ (right panels) comparisons across (A) ADNC score, (B) Thal Phase, and (C) Braak stage. Boxplots show median, interquartile range (IQR), and outliers. Color represents severity. Asterisks represent *P*‐values from Wilcoxon pairwise comparisons (*P < 0.05, **P < 0.01, ***P < 0.001, ****P < 0.0001, and not significant [ns]).

In the full autopsy sample (αSyn, αSyn+AD, AD), Figure 2 tests plasma analytes across metrics of AD severity: ADNC, Thal phase, and Braak stage.

### Associations with pathological accumulation within LBSD

**Figure 3:**
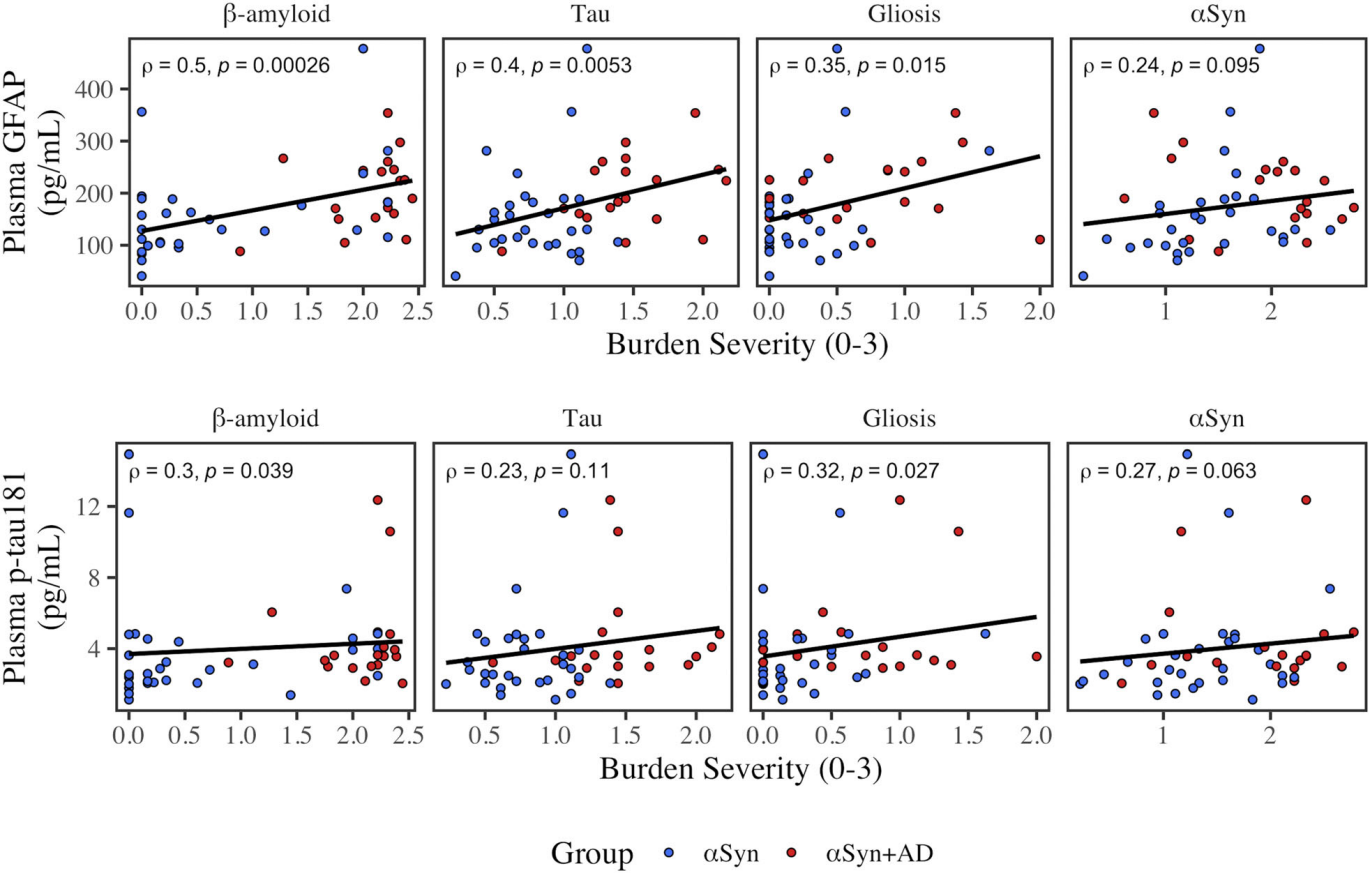
Correlations of plasma analytes and measures of pathological burden. Scatterplots for plasma GFAP (top panel) and p‐tau_181_ (bottom panel) and pathological variables are plotted. Color represents αSyn (blue) and αSyn+AD (red). Least squares regression lines are plotted in black. Spearman's rho (*ρ*) and nominal *P*‐values are reported. Only the association between plasma GFAP and β‐amyloid survived Bonferroni correction (Bonferroni‐*P* = 0.0058).

Within LBSD, we explored the pathological correlates (β‐amyloid, tau, gliosis, and αSyn) of plasma GFAP and p‐tau_181_ (Figure 3); we also tested associations with TDP‐43 and CAA to ensure other pathologies were not influencing plasma concentrations.

Across all LBSD, plasma GFAP was positively associated with β‐amyloid (*ρ* = 0.5, *P* = 0.00026; Bonferroni‐*P* = 0.0058). Associations with tau (*ρ* = 0.4, *P* = 0.0053; Bonferroni‐*P* = 0.071) and gliosis (*ρ* = 0.35, *P* = 0.015; Bonferroni‐*P* = 0.062) did not survive multiple corrections. Plasma GFAP was not associated with αSyn (*ρ* = 0.24, *P* = 0.095), TDP‐43 (*P* = 0.92), or CAA (*P* = 0.78).

Plasma p‐tau_181_ associations did not survive multiple corrections for β‐amyloid (*ρ* = 0.30, *P* = 0.039; Bonferroni‐*P* = 1.0) or gliosis (*ρ* = 0.32, *P* = 0.027; Bonferroni‐*P* = 1.0); plasma p‐tau_181_ was not associated with tau (*P* = 0.11), αSyn (*ρ* = 0.27, *P* = 0.063), TDP‐43 (*P* = 0.24), or CAA (*ρ* = 0.25, *P* = 0.079).

In addition, plasma GFAP and p‐tau_181_ were correlated with each other within LBSD (*ρ* = 0.31, *P* = 0.031).

Next, we tested how both analytes associated with AD pathological hallmarks within LBSD. A linear model tested β‐amyloid plaque as a function of both plasma p‐tau_181_ and GFAP, covarying for age at plasma collection, plasma‐to‐death interval, and sex. β‐amyloid plaque burden was significantly associated with plasma GFAP (*β* = 15, 95% CI = 6.1–25, and *P* = 0.0018) with large effect size (*η* ^2^_*G*_ = 0.23; 95% CI = 0.066–1.0), but not plasma p‐tau_181_ (*P* = 0.95). Results were robust after excluding individuals who had plasma collection >5 years before death (GFAP: *β* = 14, 95% CI = 3.7–24, and *P* = 0.0089; p‐tau_181_: *P* = 0.82).

Likewise, we tested pathological tau burden as a function of both plasma p‐tau_181_ and GFAP, covarying for age at plasma collection, plasma‐to‐death interval, and sex. Pathological tau burden was significantly associated with plasma GFAP (*β* = 12, 95% CI = 2.5–22, *P* = 0.015) with large effect size (*η* ^2^_*G*_ = 0.23; 95% CI = 0.066–1.0), but not plasma p‐tau_181_ (*P* = 0.34). Results were robust after excluding individuals who had plasma collection >5 years before death (GFAP: *β* = 12, 95% CI = 2.1–23, and *P* = 0.020; p‐tau_181_: *P* = 0.30).

β‐amyloid plaque burden was not significantly associated with any of the covariates: age (*P* = 0.99), plasma‐to‐death (*P* = 0.62), or sex (*P* = 0.10). Tau burden was not associated with age (*P* = 0.30), plasma‐to‐death (*β* = 1.5, 95% CI = −0.075 to 3.1, and *P* = 0.061), or sex (*P* = 0.81).

Pathological accumulations of β‐amyloid, tau, and gliosis were all positively correlated in LBSD (β‐amyloid and tau: *ρ* = 0.51, *P* = 0.00017; β‐amyloid and gliosis: *ρ* = 0.32, *P* = 0.025; tau and gliosis: *ρ* = 0.46, *P* = 0.001). Given that plasma GFAP was positively associated with β‐amyloid, tau, and gliosis burden (Figure 3) and thus the potential for collinearity, a post hoc linear model tested for plasma GFAP as a function of all three pathologies (β‐amyloid, tau, and gliosis burden) to determine which might independently associate with plasma GFAP levels. In addition, age at plasma, plasma‐to‐death interval, and sex were included as covariates. Plasma GFAP was significantly associated with β‐amyloid burden (*β* = 0.011, 95% CI = 0.0011–0.02, and *P* = 0.030) with large effect size (*η* ^2^_*G*_ = 0.3; 95% CI = 0.12–1.0); however, GFAP was not associated with gliosis (*P* = 0.35) or tau (*P* = 0.41) after covarying for β‐amyloid. In this model, covariates age (*β* = 0.014, 95% CI = −0.0021 to 0.030, and *P* = 0.086), plasma‐to‐death (*β* = −0.043, 95% CI = −0.090 to 0.0041, and *P* = 0.073), and sex (*P* = 0.15) were not significant.

### ROC analyses

**Figure 4:**
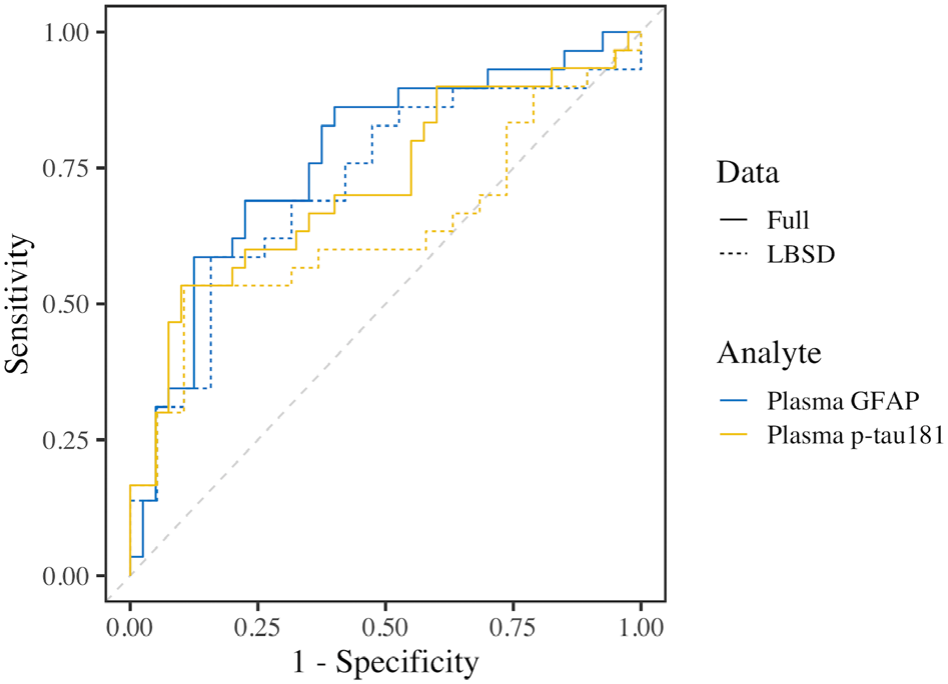
Receiver operating characteristic (ROC) curves. Color indicates plasma GFAP (blue) and p‐tau_181_ (yellow). Solid lines represent the full autopsy dataset (αSyn, αSyn+AD, and AD), and dotted lines represent LBSD αSyn‐positive cases (αSyn, αSyn+AD). The dashed gray line represents chance performance. AUCs were 0.77 for full sample GFAP, 0.71 for LBSD sample GFAP, 0.72 for full sample p‐tau_181_, and 0.64 for LBSD sample p‐tau_181_.

ROC analyses (Figure 4) tested the diagnostic accuracy of both analytes to detect ADNC (high/intermediate) in the full sample (αSyn, αSyn+AD, and AD) and again in LBSD αSyn‐positive cases (αSyn, αSyn+AD). Neither analyte demonstrated high diagnostic performance in LBSD: for the full sample, plasma GFAP had an AUC of 0.77 (90% CI = 0.67–0.86) and plasma p‐tau_181_ had an AUC of 0.72 (90% CI = 0.61–0.82). In the LBSD sample, plasma GFAP had an AUC of 0.71 (90% CI = 0.58–0.83) and plasma p‐tau_181_ had an AUC of 0.64 (90% CI = 0.50–0.77).

### Clinical correlation with MMSE

**Figure 5:**
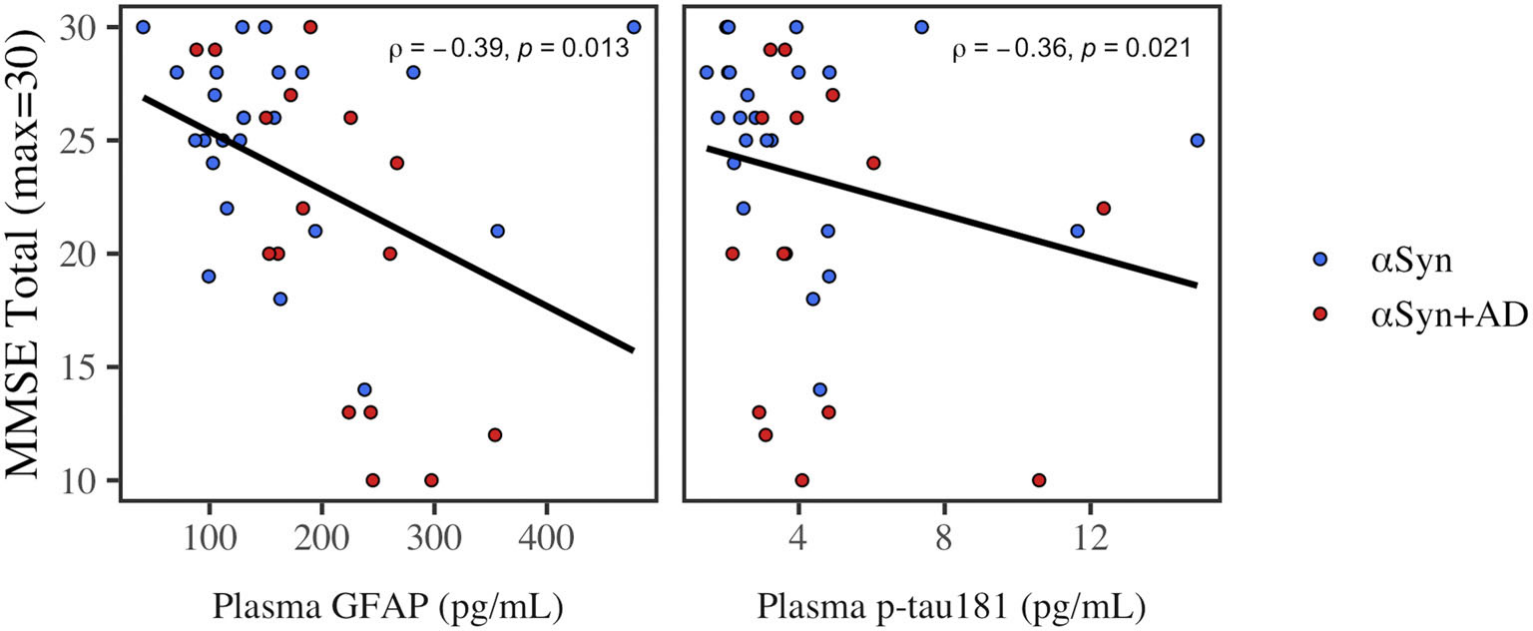
Associations between analytes and MMSE. Color indicates LBSD group αSyn (blue) and αSyn+AD (red). Least squares regression line is plotted. Spearman's rho (*ρ*) and *P*‐value are reported.

In LBSD, we tested how plasma analytes associated with cognition; Spearman's correlations show that both plasma GFAP and p‐tau_181_ were correlated with MMSE (Figure 5). In a model covarying for age, plasma‐to‐MMSE interval and sex, lower MMSE was associated with higher GFAP (*β* = −8.2, 95% CI = −1.5 to −1.2, and *P* = 0.024), but not p‐tau_181_ (*P* = 0.55). We repeated the model, excluding cases with plasma‐to‐MMSE interval >1 year: results were consistent with lower MMSE significantly associated with higher GFAP (*β* = −12, 95% CI = −2.1 to −1.8, *P* = 0.023), but not p‐tau_181_ (*P* = 0.44).

Examining covariates, MMSE was associated with plasma to MMSE interval (*β* = −2.1, 95% CI = −3.2 to −0.89, and *P* = 0.001) and sex (*β* = −7.8, 95% CI = −1.5 to −0.14, and *P* = 0.046), but was not associated with age (*P* = 0.70).

## Discussion

Accumulating evidence shows that plasma biomarkers, such as p‐tau_181_ and GFAP, are highly sensitive to AD.^12^, ^13^, ^40^ Yet, plasma biomarkers have not been well examined in the context of LBSD with concomitant ADNC. In this autopsy study, we show consistent results that plasma GFAP is sensitive to concomitant ADNC in LBSD with autopsy‐confirmed αSyn: antemortem plasma GFAP was significantly higher in αSyn+AD than αSyn, it was associated with higher burden of postmortem β‐amyloid (even after covarying for gliosis), and was associated with worse antemortem MMSE performance. Surprisingly, we do not find as robust results for plasma p‐tau_181_. Plasma p‐tau_181_ was not higher in αSyn+AD than αSyn. Postmortem pathological associations with β‐amyloid plaques and neurofibrillary tau showed a stronger association with plasma GFAP than p‐tau_181_. Our findings emphasize conclusions from previous studies that LBSD‐specific strategies may be necessary to detect concomitant AD,^8^, ^10^, ^41^ and that plasma biomarkers show unique profiles that may diverge from those seen in CSF.^42^, ^43^ Our results here suggest that plasma GFAP is a promising biomarker that is sensitive to concomitant AD pathology in LBSD, and may reflect accumulation of β‐amyloid pathology.

We note that ADNC is typically less severe in LBSD cases than clinical AD.^1^, ^2^ In this study, 74% of αSyn+AD in this sample were intermediate ADNC (26% were high ADNC). In AD, a higher percentage were high ADNC (95%), and likewise AD cases showed significantly higher plasma p‐tau_181_ than αSyn+AD. When examining ADNC, Thal phase, and Braak stage, plasma p‐tau_181_ appeared elevated only at the highest levels (i.e., high ADNC, Thal/Braak = 3); our findings echo other research showing plasma p‐tau_181_ is elevated only at more severe AD stages,^44^ and is significantly lower in intermediate ADNC than high ADNC cases.^45^ Likewise, studies that demonstrate good diagnostic accuracy of plasma p‐tau_181_ in autopsy samples have typically tested discrimination of high ADNC from intermediate/low/not ADNC, showing ROC AUCs of 0.77–0.91.^12^, ^13^, ^46^ Longitudinal studies show that this relationship may depend on time between plasma collection and death.^40^ An open question is whether other epitopes of p‐tau (such as 217 or 231) are more sensitive to intermediate ADNC than p‐tau_181_, especially at earlier AD stages,^13^, ^44^, ^45^, ^47^ including preclinical AD.^48^ If so, p‐tau 217 or 231 may be more useful for detecting concomitant AD in LBSD. Poor sensitivity of plasma p‐tau_181_ to intermediate ADNC may help explain the divergences between previous findings: one study showed no difference in plasma p‐tau_181_ in LBSD who were PET‐Aβ positive versus PET‐Aβ negative,^20^ although others have found that plasma p‐tau_181_ does associate with PET‐tau status in LBSD^21^ and that it associates with cognitive decline in DLB.^22^ Another possible factor is that biomarkers, like CSF and PET, can show altered profiles in LBSD compared to AD.^10^, ^41^ There is some rare autopsy work examining the effects of concomitant pathology on plasma biomarkers in primary AD^40^, ^45^; findings demonstrate significantly higher plasma p‐tau_181_ in primary AD patients with mixed pathology, including concomitant Lewy bodies, than non‐AD. Future studies will be needed to disentangle these contributing factors, to test if p‐tau 217 and 231 epitopes are more sensitive to intermediate ADNC than p‐tau181, and test utility of different p‐tau epitopes in LBSD to detect αSyn+AD.

Despite the high proportion of intermediate ADNC in αSyn+AD, our findings provide strong evidence that plasma GFAP is sensitive to AD‐copathology in LBSD. A robust astrocytic response to β‐amyloid plaques^49^, ^50^ may in part explain the strong links found between plasma GFAP and β‐amyloid.^16^, ^18^, ^51^ In support, we find that plasma GFAP is sensitive to pathological alterations due to concomitant AD, and is most strongly associated with postmortem β‐amyloid accumulation. Associations between GFAP and β‐amyloid remained robust even after covarying for colinear tau and gliosis burden. These findings are in accordance with previous work showing plasma GFAP is associated with PET‐Aβ and CSF Aβ_42_/Aβ_40._ ^16^, ^17^ In LBSD, β‐amyloid burden is often high and may have a synergic relationship with αSyn pathology,^52^ which might in part explain the superior performance of plasma GFAP in this study compared with plasma p‐tau_181_. Our analyses also point to the clinical relevance of elevated plasma GFAP, which was associated with impaired cognition (MMSE). While this study focuses on end‐stage disease in LBSD, it will be critical for future studies to explore whether our findings generalize to other neurodegenerative disease and other stages of disease: whether plasma GFAP is more sensitive to intermediate ADNC than p‐tau_181_ in primary AD cases, and whether plasma GFAP is elevated in early/prodromal stages of disease in AD and LBSD. Likewise, it will be important for future studies to test how GFAP changes over disease course and if it predicts future cognitive decline in LBSD.

There are several caveats to consider when interpreting our findings. First, while our focus on LBSD and AD copathology is a strength of this study, it must be noted that pathological associations with plasma biomarkers observed here may not generalize to other conditions, such as primary AD. We also acknowledge that, despite strong associations of plasma GFAP with β‐amyloid, we do not find a plasma biomarker strategy that robustly identifies concomitant ADNC in LBSD: the best ROC AUC was 0.71 using plasma GFAP. Future studies should test if plasma GFAP has added value when combined with other biomarker modalities, like CSF or PET. Second, this study focused on end‐stage disease, and tested how plasma levels closest to death associated with postmortem pathological accumulations. Because of this, some subjects had a substantial interval between plasma collection and death. To help account for this, models included plasma‐to‐death interval as a covariate and subanalyses confirmed results after excluding individuals with an interval >5 years. Still, it will be important for future studies to track longitudinal changes in plasma biomarkers within LBSD and to test plasma GFAP and p‐tau_181_ in the context of early stage LBSD. Third, plasma concentrations in LBSD may be confounded by other factors not available in this study, including body mass index (i.e., blood volume) and creatinine (i.e., kidney functioning).^53^ Fourth, we measured plasma p‐tau_181_ concentrations using an established platform that shows excellent performance in AD,^33^, ^54^ but did not test other isoforms of p‐tau. Future studies should test if other isoforms of plasma p‐tau (217 or 231) or measures from different platforms might perform differently in LBSD. Fifth, effects of race were not able to be assessed in this autopsy sample, which was majority white, and thus the generalizability of our findings are limited. It has been shown that CSF p‐tau_181_ levels are lower in Americans who are Black compared to White^55^, ^56^ and is an important factor for plasma as well.^12^ Thus, race can be an important factor in plasma levels, and future studies should test how race affects plasma p‐tau_181_ and GFAP performance in LBSD. Finally, in addition to β‐amyloid, plasma GFAP has been previously associated with white matter disease in AD.^18^, ^19^ We examined the possible influence of other pathologies but found no evidence that plasma GFAP was associated with CAA; this null association was also observed in a mixed pathology sample.^57^ Only three LBSD autopsy patients had significant cerebrovascular disease, and we were not able to assess how GFAP and p‐tau_181_ levels would be altered by moderate or high vascular disease in this study.

While many studies have tested AD plasma biomarkers p‐tau_181_ and GFAP in the context of clinical AD, findings have not been validated in LBSD with autopsy‐confirmed αSyn and AD neuropathologic diagnoses. This autopsy study focuses on LBSD to evaluate AD plasma biomarkers for detecting concomitant ADNC in αSyn cases. Analyses demonstrated that plasma GFAP was sensitive to concomitant ADNC in LBSD: plasma GFAP was higher in αSyn+AD than αSyn, was sensitive to brain β‐amyloid in LBSD, and was associated with global cognition in LBSD. Together, our findings demonstrate that plasma GFAP is associated with β‐amyloid and concomitant AD in LBSD.

## Author Contributions

KC and TT contributed to the conception and design of the study. KC, DI, AC, SA, LS, EL, DW, DW, MS, AD, MG, and TT all contributed to the methodology and design of the study. KC, DI, AC, SA, LS, EL, DW, DW, MS, AD, MG, and TT contributed to the acquisition and analysis of data. KC and TT contributed to drafting the text or preparing the figures.

## Conflict of Interest

Authors report no conflicts of interest relevant to this study.

## Data Sharing

Anonymized data will be shared by a reasonable request from any qualified investigator.

## Data Access

KAQC and TFT had full access to all the data in the study and would take responsibility for the integrity of the data and the accuracy of the data analysis.
